# Low-Frequency Electroacupuncture Improves Insulin Sensitivity in Obese Diabetic Mice through Activation of SIRT1/PGC-1*α* in Skeletal Muscle

**DOI:** 10.1155/2011/735297

**Published:** 2010-10-26

**Authors:** Fengxia Liang, Rui Chen, Atsushi Nakagawa, Makoto Nishizawa, Shinichi Tsuda, Hua Wang, Daisuke Koya

**Affiliations:** ^1^Department of Acupuncture and Moxibustion, Hubei University of Chinese Medicine, Wuhan 430061, China; ^2^Endocrinology and Metabolism Division, Kanazawa Medical University, 1-1 Daigaku, Kahoku-Gun, Ishikawa 920-0293, Japan; ^3^Department of Traditional Chinese Medicine, Union Hospital, Tongji Medical College, Huazhong University of Science and Technology, Wuhan 430022, China

## Abstract

Electroacupuncture (EA) has been observed to reduce insulin resistance in obesity and diabetes. However, the biochemical mechanism underlying this effect remains unclear. This study investigated the effects of low-frequency EA on metabolic action in genetically obese and type 2 diabetic db/db mice. Nine-week-old db/m and db/db mice were randomly divided into four groups, namely, db/m, db/m + EA, db/db, and db/db + EA. db/m + EA and db/db + EA mice received 3-Hz electroacupuncture five times weekly for eight consecutive weeks. In db/db mice, EA tempered the increase in fasting blood glucose, food intake, and body mass and maintained insulin levels. In EA-treated db/db mice, improved insulin sensitivity was established through intraperitoneal insulin tolerance test. EA was likewise observed to decrease free fatty acid levels in db/db mice; it increased protein expression in skeletal muscle Sirtuin 1 (SIRT1) and induced gene expression of peroxisome proliferator-activated receptor *γ* coactivator 1*α* (PGC-1*α*), nuclear respiratory factor 1 (NRF1), and acyl-CoA oxidase (ACOX). These results indicated that EA offers a beneficial effect on insulin resistance in obese and diabetic db/db mice, at least partly, via stimulation of SIRT1/PGC-1*α*, thus resulting in improved insulin signal.

## 1. Introduction

Obesity is a serious health issue that is prevalent worldwide. It currently affects over 396 million individuals across the globe, and this figure is expected to climb to over 573 million by 2030 [1]. Insulin resistance is characterized as the most critical factor that contributes to the development of obesity among patients afflicted with type 2 diabetes mellitus (T2DM). Thus, reduction of insulin resistance is an important clinical goal today.

In mammals, Sirtuin 1 (SIRT1) is one of the seven homologs of silent information regulator 2 (Sir2). It plays a critical role in DNA damage response, metabolism, and longevity [2]. Recent studies suggest an association between SIRT1 and insulin sensitivity [3]. SIRT1 augments insulin sensitivity by repressing inflammation and having a direct or indirect involvement in the insulin-signaling pathway [3–5]. Remarkably, SIRT1 activators enhance insulin sensitivity in vitro and ameliorate insulin resistance in vivo in a SIRT1-dependent manner [4, 6]. Moreover, overexpression of SIRT1 protects against insulin resistance in diabetic models [7] and high-fat-diet-induced metabolic disorder [8]. Taken collectively, these findings implicate SIRT1 activation as a potential therapeutic target in overcoming insulin resistance. 

Peroxisome proliferator-activated receptor *γ* (PPAR*γ*) coactivator 1*α* (PGC-1*α*) ranks among the major substrates of SIRT1. PGC-1*α* is a metabolic coactivator that interacts with transcription factors to induce mitochondrial biogenesis and respiration [9]. In human skeletal muscle, low levels of nuclear-encoded PGC-1*α* and mitochondrial-encoded gene COX1 suggest a role for impaired mitochondrial function in the development of insulin resistance [10]. High-fat-diet-induced insulin resistance occurs together with decreased muscle PGC-1*α* expression, persistent elevation in intramuscular acylcarnitines, and metabolic byproducts of incomplete fatty acid oxidation. Increased PGC-1*α* activity and/or enhanced mitochondrial efficiency may protect against lipid-induced insulin resistance [11]. Deacetylation of PGC-1*α* by SIRT1 increases mitochondrial biogenesis and activates genes associated with mitochondrial fatty acid oxidation [12]. Collectively, these findings indicate that therapy targeting SIRT1/PGC-1*α* and mitochondria may serve as a novel approach for curbing insulin resistance.

In experimental research and clinical studies, acupuncture has been observed to reduce obesity-related insulin resistance [13–15]. However, though acupuncture has the potential to improve pathological changes in the mitochondria [16], the biochemical mechanism underlying its effect on insulin resistance remains elusive. Meanwhile, electric stimulation such as exercise induces muscle contraction, which has been observed to activate SIRT1/PGC-1*α* [17, 18]. It is interesting to examine if the combination of acupuncture and electric stimulation will yield merits for the improvement of insulin sensitivity.

The present study tested the hypothesis that electroacupuncture (EA) ameliorates insulin sensitivity via regulation of SIRT1/PGC-1*α* and improving mitochondrial function. EA is a type of acupuncture wherein needles are attached to an apparatus that produces continuous electric pulses. To investigate the effect of EA on insulin resistance, this study was conducted on db/db mice, a genetic model of insulin resistance and T2DM. Low-frequency EA produced insulin-sensitizing effects and modulated free fatty acid (FFA) levels in db/db mice. Strikingly, EA likewise induced SIRT1 protein expression, which was concordant with increased PGC-1*α*, nuclear respiratory factor 1 (NRF1), and acyl-CoA oxidase (ACOX) gene expression in the skeletal muscle of db/db mice. Based on these findings, EA is proposed to improve insulin sensitivity in db/db mice, at least partly, via stimulation of mitochondrial biogenesis and lipid oxidation involving SIRT1/PGC-1*α* activation.

## 2. Materials and Methods

### 2.1. Animals

Male, seven-week-old, C57BL/KsJ-Lep^db/db^ mice (db/db mice) and their lean db/m heterozygote littermates were obtained from CLEA Japan, Inc. (Tokyo, Japan). They were housed at 22°C in a controlled environment and received 12 h of artificial light per day. They were allowed access to normal laboratory chow and water ad libitum. All experiments conducted on these samples were approved by the Animal Experimental Committee of Kanazawa Medical University.

### 2.2. Experimental-Design

After two weeks of acclimatization, the samples were randomly divided into four groups: db/m (*n* = 8), db/m + EA (*n* = 6), db/db (*n* = 8), and db/db + EA (*n* = 8). EA was applied at the acupuncture points of Zusanli (ST36) and Guanyuan (CV4) using 0.30 × 25 mm needles (Suzhou Acupuncture & Moxibustion Appliance Co, China). ST36 is located 5 mm below and lateral to the anterior tubercle of the tibia; at this point, needles were inserted perpendicularly at 3–5 mm. CV4 is located at the juncture of upper 6/7 and lower 1/7 of the line that links the xiphoid process and external genitalia; the needle at this point was inserted obliquely towards the xiphisternum at 3–5 mm. Needles at CV4 and ST36 on one side, which were linked to ST36 on the other side on the following day, were linked with two electrodes of an electrostimulator (G6805-2A, Shanghai Huayi Medical Instrument Factory, China). The points were electrically stimulated with successive low-frequency waves of 3 Hz. Intensity was adjusted to produce local muscle contractions that varied from 0.5 to 0.8 mA. db/m+EA and db/db+EA groups received EA treatment for 10 min per day, with five treatments being performed weekly. Neuronal activity was assumed to affect transmission of acupuncture stimulation; thus, the mice were not anesthetized during acupuncture. db/m and db/db mice were placed in cages used for EA treatments for the same 10-min periods. Treatment lasted for eight weeks.

### 2.3. Body Mass, Food Intake, Fasting Blood Glucose, Plasma Insulin, and HbA1c

Body mass, food intake, and fasting blood glucose (FBG) were analyzed at zero, two, four, six, and eight weeks after commencement of EA treatment. Tail-snip fasting glucose levels were measured using a glucose testing machine and corresponding cartridge (Antesense III from Horiba, Japan). After two and eight weeks of treatment, tail blood was collected to assay plasma fasting insulin (1,000 g for 15 min at 4°C) using a commercial enzyme-linked immunosorbent assay (ELISA) kit (ARKIN-011T, Shibayagi, Japan). Plasma HbA1c levels were measured using an automatic glycohemoglobin analyzer ADAMS A1c HA-8160 (Arkray Inc., Kyodo, Japan).

### 2.4. Intraperitoneal Insulin Tolerance Test and Intraperitoneal Glucose Tolerance Test

Intraperitoneal insulin tolerance tests (IPITTs) were performed after six weeks of EA treatment. After 12 h of fasting, an insulin solution of 2 U/kg of body mass was injected intraperitoneally into the mice; blood samples were collected for glucose determination prior to insulin administration and after 15, 30, 60, and 90 min. Intraperitoneal glucose tolerance tests (IPGTTs) were performed seven weeks following the series of treatments. Meanwhile, mice that were allowed to fast for 12 h received an intraperitoneal injection of glucose (1 mg glucose/g body mass), and blood samples were collected for glucose level determination at zero, 15, 30, 60, and 120 min following glucose injection. After insulin or glucose administration, blood glucose was assayed from 10 *μ*L of blood collected from the tip of the tail vein.

### 2.5. Serum FFA, Triglyceride, Total Cholesterol, and Corticosterone

After the treatment, blood was collected from the inner canthus using a capillary, and it was centrifuged at 1,000 g for 15 min at 4°C. The resultant serum was stored at −20°C prior to analysis. Serum FFA or nonesterified fatty acid, NEFA (ACS-ACOD method), triglyceride or TG (GPO-DAOS method), and total cholesterol or TC (DAOS method) were assayed using respective kits (Wako Pure Chemical Industries, Japan). Serum corticosterone levels were measured using corticosterone enzyme immunoassay (EIA) kit (Beckman Coulter, Inc. USA, REF: DSL-10-81100).

### 2.6. Real-Time Reverse Transcriptional Polymerase Chain Reaction

Mice were sacrificed at the end of the treatment. Excised quadriceps muscle tissues were stored overnight at 4°C in RNAlater solution (Qiagen Inc., Tokyo, Japan), and subsequently at −20°C prior to total RNA extraction. This was conducted following the method described in a previous work [19].

RNA concentrations were determined at the 260/280 nm absorbance ratio. An aliquot (1 *μ*g) of extracted RNA was reverse transcribed into first-strain complementary DNA (cDNA) using a PrimeScript RT reagent Kit (Perfect Real Time, Takara Code RR037A, Japan) following the instructions provided by the manufacturer. The following thermal cycling protocol was used for reverse transcription: 30°C for 10 min, 42°C for 45 min, and 99°C for 5 min. It was then stored at 4°C.

Real-time reverse transcriptional polymerase chain reaction (RT-PCR) was performed with a 7700 Real-Time RT-PCR system (ABI PRISM, 7700 Sequence Detector) using the DNA-binding dye SYBR green to detect PCR products. The reaction mixture contained SYBR Green Master Mix 10 *μ*L (Toyobo Company Ltd., Osaka, Japan), 2 *μ*L enhancer, 0.8 *μ*L custom-synthesized primers (forward and reverse primers, 10 *μ*M), and cDNA equivalent to 20 ng total RNA in a final reaction volume of 20 *μ*L. PCR protocol included initial denaturation of 10 s at 50°C, followed by 32 cycles of amplification for 5 min at 95°C, 15 s at 95°C, and 1 min at 60°C. Duplicate samples were run for real-time RT-PCR, and amplification products were qualified using a standard calibration curve. Relative expression was calculated as follows: density of the product of respective target gene divided by that for GAPDH from the same cDNA. Specific primers used for PCR are listed in Table 1.

### 2.7. Western Blotting

A total of 100 mg quadriceps tissue sample was homogenized in 1 mL ice-cold lysis buffer (2% lithium lauryl sulfate (LDS), 1 v/v% 1.7 mg/mL aprotinin, 1 v/v% 10 mg/mL phenylmethylsulfonyl fluoride (PMSF), and 1 mM sodium orthovanadate). The homogenate was centrifuged at 15,000 rpm for 15 min at 4°C. Supernatants were collected, and protein concentrations were determined using a bicinchoninic acid protein assay kit (Pierce Biotechnology, USA, 1859078). Supernatants were stored at −80°C prior to d use.

Equivalent amounts of protein for each sample were incubated at 95°C for 5 min in sample buffer. Subsequently, these were electrophoretically separated on 10% sodium dodecyl sulfate- (SDS-) polyacrylamide gels (Atto Corporation, Tokyo, Japan) prior to being transferred onto PVDF membrane (Pall Corporation). Nonspecific reactivity was blocked in 5% nonfat dry milk in PBST (10 mM Tris-HCl, pH 7.5, 150 mM NaCl, 1% Tween-20) for 1 h at 6–8°C. Afterwards, the membrane was incubated overnight at 4°C with anti-SIRT1 rabbit antibody (07-131; 1 : 1000; Upstate Biotechnology, Lake Placid, NY, USA). An antibody that recognizes *α*/*β*-tubulin (no. 2148, 1 : 1000; Cell Signaling Technology) was utilized as a reference. 

Stabilized goat antirabbit IgG HRP-linked antibody (no. 7074, Cell Signaling Technology) or antimouse IgG HRP-linked antibody (no. 7076, Cell Signaling Technology) was used as secondary antibody. Bands were visualized using an enhanced chemiluminescence Western blotting analysis system (no. 34095, PIERCE) and luminescent image analyzer (LAS-4000, Fujifilm Corporation, Tokyo, Japan). Densitometry was performed using NIH Image J software. SIRT1 immunoreactivity was normalized against the *α*/*β*-tubulin result. The experiment was repeated at least thrice for each protein in each sample.

### 2.8. Statistical Analysis

Data were expressed as mean ± SE. Trapezoidal rule was used to determine area under the IPGTT curve (AUCg). Meanwhile, analysis of variance (ANOVA) with subsequent Bonferroni's test was employed to determine the significance of differences in multiple comparisons. A *P* value of less than .05 was considered statistically significant.

## 3. Results

### 3.1. FBG Decreased and Fasting Plasma Insulin Levels Were Maintained by EA

At nine weeks of age, the db/db mice exhibited hyperglycemia compared to their db/m littermates. It was observed that EA treatment lasting two weeks was suitable for lowering FBG of db/db mice. After six weeks of treatment, FBG levels decreased significantly in EA-treated db/db mice compared with untreated db/db littermates; the effect became more significant after eight weeks of treatment (Table 2). EA produced no significant effect on the FBG of db/m mice compared with untreated db/m mice.

Compared to their db/m littermates, db/db mice exhibited hyperinsulinemia at 11 weeks of age (Table 2). After two weeks of treatment, improved insulin sensitivity following EA treatment was demonstrated by reduced insulin levels in EA-treated mice that were subjected to overnight fasting. However, after eight weeks of treatment, plasma insulin levels in EA-treated db/db mice that experienced fasting were significantly higher than those of untreated db/db mice. Further, plasma fasting insulin levels in 17-week-old untreated db/db mice were significantly decreased compared with untreated db/db mice at 11 weeks of age.

### 3.2. Body Mass Gain and Food Intake Were Reduced

Body mass of db/db mice was higher than their db/m littermates at nine weeks of age. This continued to increase to nearly twice that of db/m mice by 17 weeks (Table 2). Low-frequency EA induced a significantly reduced body mass gain among db/db mice after six weeks of treatment.

Food intake of db/db mice was higher by 1.4 folds to two folds compared with that of db/m mice throughout the experiment period. EA reduced food intake of db/db mice significantly after six weeks of treatment (Table 2).

### 3.3. Plasma HbA1c Levels Were Not Affected

Plasma HbA1c levels were measured to investigate the long-term effect of EA on glucose metabolism. At 17 weeks, db/db mice displayed markedly higher plasma HbA1c levels compared with db/m mice. EA treatment induced a decrease in plasma HbA1c levels in db/db mice compared with non-EA-treated db/db mice (Table 2), through in the absence of statistical significance (*P* = .053).

### 3.4. EA Decreased Serum FFA, with No Significant Effect on TC, TG, or Corticosterone Levels

Blood glucose control may be attributed to improved insulin sensitivity; this may result in reduced blood lipid levels as well. Serum FFA, TC, and TG levels were elevated in db/db mice compared with db/m mice. EA treatment caused a significant decrease in FFA concentrations in db/db mice compared with untreated littermates (Table 2). A slight, though insignificant, decrease in TC and TG was observed as well (Table 2). EA produced no effect on FFA, TC, or TG in db/m mice compared with untreated db/m controls.

At the end of treatment, serum corticosterone levels were measured to evaluate potential stress induced by treatment. As demonstrated in a previous study [20], db/db mice displayed higher corticosterone levels than their littermates (Table 2). EA treatment did not affect serum corticosterone of db/m or db/db mice, indicating that handling and treatment were not stressful for the subjects.

### 3.5. EA Improved IPITT, with No Significant Impact on IPGTTs and AUCg

**Figure 1:**
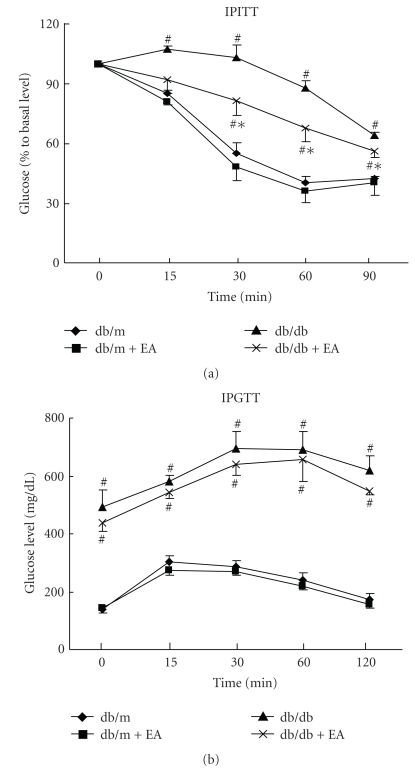
Effect of electroacupuncture on IPITTs and IPGTTs. (a) Intraperitoneal insulin tolerance test. Mice were fasted overnight and then injected with insulin solution (2 U/kg of body mass) intraperitoneally. Blood glucose levels were determined at the time points indicated. (b) Intraperitoneal glucose tolerance test. Mice were fasted overnight and then injected intraperitoneally with glucose (1 mg glucose/g of body mass). Blood glucose levels were measured at the indicated time points. Each data point represents the mean ± SE of four mice. ^#^ *P* < .05 versus db/m and db/m+EA, *P < .05 versus db/db.

Based on insulin tolerance testing, it was observed that the glucose-lowering effects of insulin were higher in EA-treated db/db mice compared with untreated littermates (Figure 1(a)). IPGTTs suggested that glucose tolerance did not differ significantly between EA-treated and -untreated db/db mice (Figure 1(b)). AUCg data revealed a slight decrease, without significance, in EA-treated db/db mice compared with untreated controls (Table 2).

### 3.6. EA Increased SIRT1 Protein Expression, Producing No Effect on SIRT1 mRNA Expression

**Figure 2:**
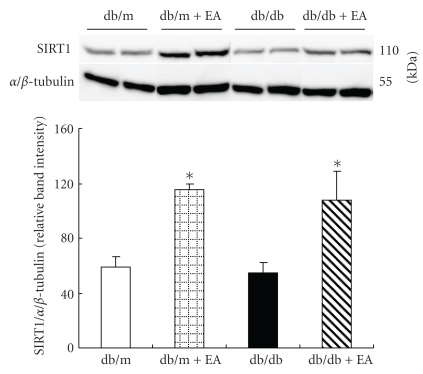
Effect of electroacupuncture on SIRT1 protein expression in skeletal muscle of nondiabetic db/m and diabetic db/db mice. Electroacupuncture increased SIRT1 protein expression in both groups. Total protein obtained from quadriceps muscles of the mice was subjected to western blotting for SIRT1. *α*/*β*-tubulin was used as a reference protein. Data are shown as the mean ± SE of four mice in each group. *P < .05 versus db/m and db/db.

**Figure 3:**
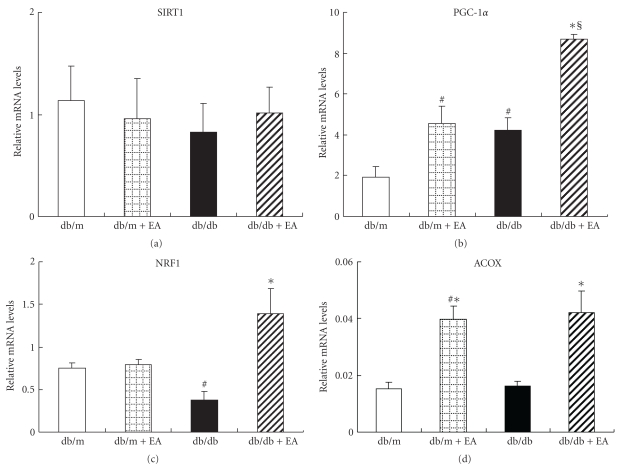
Effect of electroacupuncture on SITR1, PGC-1*α*, NRF1, and ACOX gene expressions in skeletal muscles of nondiabetic db/m and diabetic db/db mice. (a) Electroacupuncture had no significant effect on SIRT1 mRNA levels in db/m and db/db mice. (b) Electroacupuncture upregulated PGC-1*α* mRNA levels in both db/m and db/db mice. (c) Electroacupuncture increased NRF1 mRNA levels in db/db mice. (d) Electroacupuncture increased ACOX mRNA levels in db/m and db/db mice. Quadriceps muscles of the mice were collected for mRNA expression, which was estimated using quantitative real-time RT-PCR and normalized to the expression of GAPDH. Graph shows the percentage of mRNA relative to GAPDH in each group. Each value represents the mean ± SE of four mice. ^#^ *P* < .05 versus db/m, *P < .05 versus db/db, and ^§^ *P* < .01 versus db/m.

The effect of EA on SIRT1 gene expression and protein levels was investigated in view of SIRT1's association with metabolic activity and its critical role in insulin sensitivity. EA significantly increased SIRT1 protein levels in db/db and db/m mice (Figure 2), but it produced no significant effect on SIRT1 mRNA levels (Figure 3(a)). This indicates that SIRT1 may be regulated posttranscriptionally. This is supported by a recent demonstration that SIRT1 levels were posttranscriptionally modified by phosphorylation of cell cycle-dependent kinase Cdk1 [21].

### 3.7. PGC-1*α*, NRF1, and ACOX mRNA Expressions Were Upregulated

Transcriptional coactivator PGC-1*α* is crucial for mitochondrial biogenesis and fatty acid oxidation. To detect the effect of EA on mitochondrial biogenesis, PGC-1*α* gene expression in skeletal muscle was analyzed. The db/db mice exhibited significantly increased PGC-1*α* mRNA expressions compared with the db/m controls (Figure 3(b)); this observation is in agreement with a previous study [22]. EA resulted in modest upregulation of PGC-1*α* mRNA (2-3-fold), which is similar to the effect of Pioglitazone on the induction of skeletal muscle PGC-1*α* in db/db mice [23]. 

NRF1 is a key target of PGC-1 during mitochondrial biogenesis [24]. NRF1 gene expression in the skeletal muscle of db/db mice decreased significantly compared with db/m mice, whereas it increased by two folds to four folds in EA-treated db/db mice compared with the expression in untreated littermates (Figure 3(c)). 

ACOX, an enzyme involved in the first step of peroxisomal fatty acid oxidation pathway, was analyzed to determine the fatty acid oxidation capability of skeletal muscle. In db/db mice, it was observed that EA significantly increased ACOX gene expression (Figure 3(d)).

## 4. Discussion

Originating from China thousands of years ago, acupuncture is now widely practiced in both Eastern Asia and Western countries for treatment of a variety of human diseases, including dental pain, fibromyalgia, and knee osteoarthritis. Recently, numerous reports have proposed its application on diseases related to insulin resistance such as obesity and diabetes [13–15]. 

This study extended such previous investigations, demonstrating that low-frequency electroacupuncture could improve insulin sensitivity in db/db mice, a genetically obese diabetic animal. More importantly, this study suggested a potential molecular mechanism whereby EA treatment ameliorates insulin resistance in db/db mice. EA increased SIRT1 protein expression and upregulated PGC-1*α*, NRF1, and ACOX gene expression. In turn, this could enhance mitochondrial biogenesis and fatty acid oxidation and upregulate insulin-associated signal transduction with subsequent improvement in insulin resistance. 

Stimulation with needles from different point locations activates muscle afferents to the spinal cord and the central nervous system. EA induces the frequency-dependent release of neuropeptides [25]. Low-frequency EA (1–15 Hz) releases a sizeable number of neuropeptides, and this appears to be essential for inducing functional changes in different organ systems. More importantly, low-frequency EA is applied more frequently for the treatment of insulin resistance with beneficial results [14, 15]. Indeed, early insulin resistance in obesity is closely associated with overactivity of the sympathetic nervous system, which induces a proinflammatory state and thus contributes to the development of T2DM [26]. 

Low-frequency EA at the points of abdomen and/or hindlimb attenuates sympathetic nerve activity [27, 28], whereas EA at the points of upper limbs induces sympathetic nerve activity [29]. Therefore, this study targeted ST36 points in the hindlimb and CV4 points in the abdomen and stimulated these with low-frequency EA. 

Lines of evidence have demonstrated that EA is capable of improving hyperglycemia in the fasting stage, with a marked increase in plasma insulin levels in diabetic rats [14, 30]. In accordance with these studies, the present work has demonstrated that eight-week EA treatment decreased FBG levels and maintained insulin levels. This supports the suggestion that the effect of EA in regulating BG may be insulin dependent. 

Ameliorated insulin sensitivity after EA was established by IPITT, which may be attributed to improvement of responsiveness to insulin via excitation of somatic afferent fibers by EA [31]. Additionally, this study indicated that EA decreased HbA1c in the absence of statistical significance, which may be ascribed to insufficient course of treatment or limited quantity of subjects. Further, long-term study is necessary to warrant the effect of EA on HbA1c in more experimental animals. 

SIRT1 levels may increase in rodent and human tissues in response to calorie restriction and exercise [2]. This increase is assumed to cause favorable changes in metabolism. Indeed, activation of SIRT1 has been implicated as potential therapy to protect against insulin resistance [6, 32]. The present study revealed that EA activated SIRT1, indicating that improved insulin resistance by EA may be attributed to enhanced SIRT1 expression. Further, SIRT1 can protect against insulin resistance by deacetylating the substrate PGC-1*α* and increasing PGC-1*α* activity [33]. PGC-1*α* was recently demonstrated to integrate insulin signaling, mitochondrial regulation, and bioenergetic function in skeletal muscle [23]. Overexpression of PGC-1*α* rescued insulin signaling and mitochondrial bioenergetics, and its silencing concordantly disrupted these activities [23]. Collectively, these studies support the possibility that EA improves insulin sensitivity, at least partially, because of increasing SIRT1/PGC-1*α* in skeletal muscle. 

Intriguingly, PGC-1*α* gene expression levels of db/db mice were higher than those of db/m mice. It is possible that elevated PGC-1*α* was a compensatory response to elevated fatty acid substrate availability and reactive oxygen species (ROS) stimulation under the oxidative stress of diabetes. Alternatively, the effect may reflect the posttranslational regulation of PGC-1*α*, in which case gene expression may not always correlate with protein levels [34]. To support this, db/db mice that develop hyperglycemia have recorded lower skeletal muscle PGC-1*α* levels [23] and high PGC-1*α* mRNA levels [20] compared with strain-matched C57BL/6J mice. In this respect, the effect of EA on PGC-1*α* protein expression requires further investigation. 

**Figure 4:**
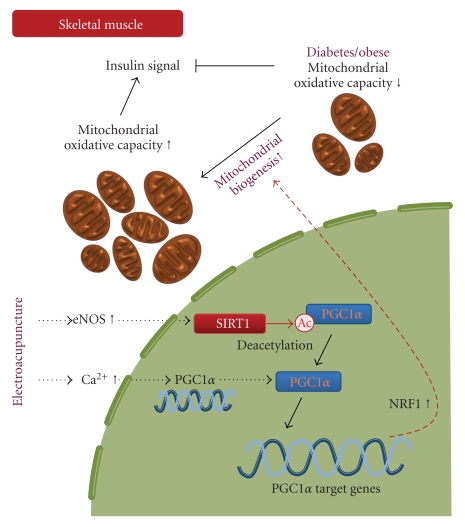
Schematic model of electroacupuncture on insulin resistance in skeletal muscle. SIRT1-mediated deacetylation of PGC1*α* is required to activate genes that are associated with mitochondrial fatty acid oxidation in response to energy demands. The resultant increase in expression of mitochondrial genes, including NRF1, could exert positive effects on insulin signaling. eNOS: endothelial nitric oxide synthase; PGC1*α*: peroxisome proliferator-activated receptor *γ* coactivator 1*α*; SIRT1: Sirtuin 1; NRF1: nuclear respiratory factor 1.

As PGC-1*α* is a coactivator for NRF1 expression [24], discrepancy between induced PGC-1*α* and reduced NRF1 gene levels in db/db mice may indicate that mitochondrial function was improved by EA [34]. The resultant increase in expression of mitochondrial genes, including NRF1, may exert positive effects on insulin signaling [12] (Figure 4).

This study has its share of limitations. There is no definite confirmation that EA improves glucose clearance and uptake into skeletal muscle to account for ITT data. Therefore, it remains a possibility that the liver, adipose tissues, or certain tissues are responsible for ITT improvement (e.g., electroacupuncture improved P-AMPK in white adipose tissue and liver; P-Akt improved P-AMPK in white adipose tissue but not in liver; data not shown). 

This study suggested a preliminary mechanism of electroacupuncture. Specifically, low-frequency EA improved insulin sensitivity in a mouse model of genetic insulin resistance and diabetes, at least in part, via stimulation of SIRT1/PGC-1*α* in the skeletal muscle. Events involved in this mechanism are presented in Figure 4. This effect leads to a net switch in the metabolic program of the organism to an adaptation that may be of benefit in the face of disorders characterized by insulin resistance. The study introduces an effective and safe activator (electroacupuncture) for SIRT1, offering a basis for applying acupuncture in clinical practice in the treatment of diseases related to insulin resistance.

##  Conflict of Interests

All authors declare that there is no conflict of interests.

## Figures and Tables

**Table 1:** Primers used in PCR.

Gene	Primer sequence	Gene number	Product length
SIRT1 (mouse, rat)	Fw 5′-CAGTGTCATGGTTCCTTTGC-3′	AF214646	104 bp
	Rv 5′-CACCGAGGAACTACCTGAT-3′		

PGC-1alpha (mouse, rat)	Fw 5′-ATGAATGCAGCGGTCTTAGC-3′	AF049330	174 bp
	Rv 5′-TGGTCAGATACTTGAGAAGC-3′		

NRF1 (mouse)	Fw 5′-GGAGCACTTACTGGAGTCC-3′	NM010938	143 bp
	Rv 5′-CTGTCCGATATCCTGGTGGT-3′		

ACOX (mouse)	Fw 5′-GGTGGTATGGTGTCGTACTTGA-3′	NM015729.2	296 bp
	Rv 5′-GAATCTTGGGGAGTTTATCTGC-3′		

GAPDH (mouse, rat)	Fw 5′-GCCAAAAGGGTCATCATCTC-3′	BC082592	226 bp
	Rv 5′-GGCCATCCACAGTCTTCT-3′		

**Table 2:** Animal characteristics and blood analyses.

Parameter (unit)	db/m (*n* = 8)	db/m+EA (*n* = 6)	db/db (*n* = 8)	db/db+EA (*n* = 8)
FBG (mg/dl)				

0w	78.8 ± 2.8	75.2 ± 2.7	149.2 ± 13.8*	156 ± 21.6*
2w	72.5 ± 2.02	64.75 ± 2.06	406.5 ± 25.40*	365.5 ± 19.25*
4w	167 ± 19.7	141.8 ± 6.2	507.8 ± 88.5*	371 ± 32.3*
6w	114.5 ± 9.1	108.2 ± 5.9	466 ± 44.1*	330 ± 28.8^∗†^
8w	105.5 ± 5.07	83.5 ± 7.5	385 ± 34.15*	282 ± 31.5^∗§^

Insulin (ng/ml)				

2w	0.53 ± 0.05	0.54 ± 0.03	3.69 ± 0.40*	2.05 ± 0.24^∗†^
8w	0.53 ± 0.05	0.54 ± 0.03	2.07 ± 0.31*	3.78 ± 0.53^∗†^

Body mass (g)				

0w	26.46 ± 0.36	26.70 ± 0.28	37.90 ± 0.68*	37.59 ± 0.14*
2w	27.78 ± 0.45	26.32 ± 0.40	42.90 ± 0.56*	40.95 ± 0.82*
4w	28.35 ± 0.57	27.65 ± 0.34	45.31 ± 0.52*	43.80 ± 0.82*
6w	29.37 ± 0.80	28.79 ± 0.78	49.01 ± 0.60*	45.21 ± 1.16^∗†^
8w	30.7 ± 0.55	28.28 ± 0.46	49.12 ± 0.63*	44.95 ± 1.45^∗†^

Food intake (g/day)				

0w	4.27 ± 0.13	4.81 ± 0.17	8.27 ± 0.72*	7.51 ± 1.02*
2w	3.94 ± 0.11	4.19 ± 0.09	7.93 ± 0.62*	7.41 ± 0.63*
4w	4.38 ± 0.12	3.91 ± 0.08	7.72 ± 0.35*	7.08 ± 0.38*
6w	3.97 ± 0.13	4.06 ± 0.12	7.27 ± 0.24*	6.45 ± 0.25^∗†^
8w	4.42 ± 0.13	4.41 ± 0.12	7.60 ± 0.23*	6.37 ± 0.30^∗†^
HbA1c (%)	3.56 ± 0.09	3.73 ± 0.12	7.55 ± 0.57*	7.03 ± 0.56*
FFA (*μ*Eq/L)	0.36 ± 0.03	0.36 ± 0.07	0.76 ± 0.04*	0.59 ± 0.03^∗†^
Triglycerides (mg/dL)	73.1 ± 14.2	66.3 ± 4.1	120.6 ± 21.9*	96.2 ± 8.3*
Cholesterol (mg/dL)	98.3 ± 3.9	90.4 ± 5.4	147.3 ± 15.15*	131.1 ± 2.8*
Corticosterone (ng/mL)	640.3 ± 137.8	773.7 ± 92.0	1154.7 ± 153.8*	1293.7 ± 81.8*
AUCg	902 ± 64.7	832 ± 24.6	2205 ± 156*	2069 ± 140*

Data are mean ± SE. *P < .05 versus db/m and db/m+EA; ^†^ *P* < .05 versus db/db; ^§^ *P* < .01 versus db/db. HbA1c: glycosylated hemoglobin A1c, FFA: free fatty acids, AUCg: area under the IPGTT (intraperitoneal glucose tolerance test) curve.
